# It’s the thought that counts: belief in suicide as an escape moderates the relationship between emotion dysregulation and suicidal ideation cross-sectionally and longitudinally

**DOI:** 10.1186/s40479-019-0112-5

**Published:** 2019-11-05

**Authors:** Nadia Al-Dajani, Amanda A. Uliaszek, Kevin Hamdullahpur

**Affiliations:** Department of Psychological Clinical Science, University of Toronto Scarborough, 1265 Military Trail, Toronto, ON M1C 1A4 Canada

**Keywords:** Suicidal ideation, Emotion dysregulation, Suicide as an escape, Moderation, Longitudinal

## Abstract

**Background:**

Previous research has illustrated a relationship between emotion dysregulation and suicidal ideation, both cross-sectionally and longitudinally. However, it is not yet understood how this relationship manifests. The aim of this study was to explore if two beliefs about suicide, (1) suicide as a way to escape from emotional pain and (2) suicide as a solution to a problem, moderate the relationship between emotion dysregulation and suicidal ideation.

**Methods:**

One hundred one community participants completed questionnaires examining emotion dysregulation, suicidal ideation, and beliefs in the functions of suicide. Inclusion criteria were used to over-sample individuals within the community experiencing higher levels of suicidal ideation and emotion dysregulation. Hierarchical linear regressions with interaction terms were used to assess moderation effects. The moderating role of beliefs in the function of suicide was examined both cross-sectionally and longitudinally.

**Results:**

Suicide as an escape significantly moderated the relationship between global emotion dysregulation and suicidal ideation cross-sectionally, while it moderated the relationship between a facet of emotion dysregulation and suicidal ideation longitudinally. Greater endorsement of this belief resulted in a stronger relationship between emotion dysregulation and suicidal ideation. The function of suicide as a solution to a problem did not moderate the emotion dysregulation-suicidal ideation relationship.

**Conclusions:**

These findings underscore the importance of not only addressing emotion dysregulation but also addressing the underlying belief that suicide is an escape in individuals who experience both emotion dysregulation and suicidal ideation. Intervention efforts to address belief in suicide as an escape along with emotion dysregulation are delineated.

## Background

Suicidal ideation is defined as thoughts of engaging in suicidal behaviour, a desire to engage in such behaviour, and/or the planning of suicide attempts [1, 2]. Onset of suicidal ideation within 1 year or suicidal ideation that includes planning is predictive of heightened risk of suicide attempt [3]. Not only can suicidal ideation translate into a behavioural risk of a suicide attempt, but it is likely to present as a chronic symptom of distress in and of itself, with at least one study finding that more than one third of individuals with suicidal ideation continued to experience intermittent thoughts over a 10-year period [4]. Research examining vulnerability factors for suicidal ideation has implicated a variety of factors, including childhood adversity [5], social isolation [6], emotion dysregulation [7]; and stressful and traumatic life events [8, 9]. The present study seeks to extend knowledge on the link between emotion dysregulation and suicidal ideation by examining potential cognitive mechanisms in this relationship.

Theoretical notions regarding the functions of suicidal ideation are not new. Baumeister [10] and Shneidman [11] both theorized that suicide could be considered the ultimate escape from extreme emotional pain. Their theory is bolstered by research illustrating a relationship between experiential avoidance (i.e., the tendency to avoid/escape painful emotions) and suicidal ideation [12–14]. Another theory suggests that suicide could be considered a solution to the problem of emotional pain if one is unable to consider other, more adaptive, solutions when facing stressful life events [15–17]. This theory has also received empirical support, with research illustrating a relationship between problem solving deficits and suicidal ideation [18–20]. It would be reasonable to hypothesize that these functions of suicide are not completely independent as both are linked to emotional pain. Yet there might be some important differences between both functions of suicide that could result in distinct processes. More specifically, escape from pain could be considered an avoidant approach while problem solving could be considered an active one, suggesting that these beliefs might be indicative of distinct deficits and might also have distinct neurobiological mechanisms. Clinically, these beliefs would likely be addressed using disparate treatment approaches. Belief in suicide as an escape from emotional pain might be best treated with interventions focused on emotional experiencing, mindfulness strategies, and acceptance of in-the-moment pain. Belief in suicide as a solution to a problem, however, might benefit more from behavioural strategies targeting problem-solving skills (e.g., brainstorming, pros and cons list) and cognitive restructuring targeting beliefs about problem-solving ability. In this study, we specifically examine if individuals who believe that suicide is the ultimate escape from emotional pain and/or if individuals who believe that suicide is a solution to a problem respond to emotional distress with suicidal ideation.

As mentioned previously, emotion dysregulation is a risk factor for thinking of suicide. Both theories on the function of suicide rest on the notion that a given instance of suicidal ideation is initially triggered by intense negative emotional arousal and a difficulty regulating that emotion in an adaptive way. Emotion dysregulation is defined as the inability to control strong negative emotions and/or the inability to accept one’s emotional experiences [21]. There is an extensive body of literature examining the relationship between emotion dysregulation and suicidal ideation, with the majority of studies using the Difficulties in Emotion Regulation Scale (DERS, [21]). The DERS is an emotion dysregulation self-report measure consisting of six subscales: (a) Nonacceptance of Emotional Responses (e.g., *When I’m upset, I feel guilty for feeling that way*); (b) Difficulties Engaging in Goal-Directed Behaviour (e.g., *When I’m upset, I have difficulty focusing on things*); (c) Impulse Control Difficulties (e.g., *When I’m upset, I lose control over my behaviours*); (d) Lack of Emotional Awareness (e.g., *When I’m upset, I* [do not] *acknowledge my emotions*); (e) Limited Access to Emotion Regulation Strategies (e.g., *When I’m upset, my emotions feel overwhelming*); and (f) Lack of Emotional Clarity (e.g., *I have difficulty making sense out of my feelings*). Cross-sectional associations between suicidality and several facets of emotion dysregulation have been previously identified across studies [22, 23]. In one longitudinal study, baseline scores on the Limited Access to Emotion Regulation Strategies scale were found to predict higher levels of suicidal ideation at follow-up [7]. Further, Wolff et al. [24] found that emotion dysregulation at baseline predicted suicidal ideation trajectory and Nonacceptance of Emotional Responses and Limited Access to Emotion Regulation Strategies differentiated chronic ideators from those with declining levels of suicidal ideation. Researchers have also found a relationship between emotion dysregulation and suicide attempts [23, 25, 26], underscoring the importance of understanding mechanisms underlying the emotion dysregulation-suicidal ideation relationship.

In this study, we examined the relationship between emotion dysregulation and suicidal ideation. We then examined whether holding the belief that a) suicide is an escape from emotional pain or b) suicide is a solution to one’s problem increased the strength of the emotion dysregulation-suicidal ideation relationship. Although we explore these beliefs as separate, we also contend that an individual can hold both beliefs about suicide in tandem. Indeed, it is possible that each belief has a similar effect on the relationship between emotion dysregulation and suicidal ideation. As such, we do not intend to pit these beliefs against each other, but rather to explore their separate effects on the relationship between emotion dysregulation and suicidal ideation. But first, we determined if these beliefs were indeed distinct enough that they be tested separately. We did this by examining the strength of the correlation between these two beliefs in our sample. If endorsed differently, we included them as separate moderators in separate models. If not, a combined score would be used as our moderator. For our moderator analyses, we hypothesized that individuals who endorsed either of these beliefs strongly would illustrate a greater relationship between emotion dysregulation and severity of suicidal ideation than those who did not endorse either belief strongly. We also explored these relationships within the specific emotion dysregulation facets.

Further, we were interested in assessing if baseline beliefs about suicidal ideation moderated the relationship between baseline emotion dysregulation and baseline suicidal ideation, and/or between baseline emotion dysregulation and ~ 6-month suicidal ideation. Theoretically, one might suspect that the impact of holding these beliefs is most pronounced for current levels of emotion dysregulation and suicidal ideation, for these beliefs might be activated in context-specific situations that are sensitive to change across time. On the other hand, one might theorize that these beliefs are longstanding and might therefore continue to moderate these relationships across time. We sought to explore both assertions.

## Methods

### Participants

Participants were recruited through advertisements placed in universities, counseling centers, and online platforms in a large metropolitan city. It was required that participants endorse experiencing three symptom criteria of borderline personality disorder (BPD), in an effort to recruit individuals with higher levels of emotion dysregulation and suicidal ideation from the community. Exclusion criteria included the presence of active psychosis or severe cognitive limitation. Phone screening was conducted by research assistants to ensure that individuals were eligible for the study. Research assistants used an adapted version of the Structured Clinical Interview for DSM-IV [27] to examine self-endorsement of three BPD symptom criteria and to investigate the presence of active psychosis. A total of 101 people participated in this study. Participants had a mean age of 27.52 (*SD* = 10.17, range 17–68) and were 62% female. 18.8% of individuals reported currently receiving some form of therapeutic support and 24.8% reported previous hospitalization due to psychological reasons. 76% of this sample reported that there is a chance that they will consider suicide in their lifetime, 51% reported there is a chance they will consider suicide in the next year, and 36% reported there is a chance they will consider suicide in the next 4 weeks. The sample had the following ethnoracial breakdown: Black/African (16%); White/Caucasian (49%); South Asian (12%); South East/Eastern Asian (17%); West Asian (Middle Eastern; 1%); Hispanic (1%); and Other (4%). Another 1% of the sample also reported that they are South Asian, 4% reported they are also Hispanic, and 1% reported they are also Aboriginal. The majority of participants reported that they were single, never married (81.2%) at the time of the assessment.

### Measures

#### The difficulties in emotion regulation scale (DERS, [21])

The DERS is a 36-item self-report questionnaire that assesses clinically relevant emotion dysregulation. The DERS is composed of six subscales: Nonacceptance of Emotional Responses (α = 0.88), Engaging in Goal-Directed Behaviour (α = 0.79), Impulse Control Difficulties (α = 0.82), Lack of Emotional Awareness (α = 0.71), Limited Access to Emotion Regulation Strategies (α = 0.79), and Lack of Emotional Clarity (α = 0.78). Each item is rated on a five-point Likert scale, ranging from *almost never* to *almost always.* Internal consistency for the DERS total score was high, with a Cronbach’s α of 0.91.

#### The Beck scale for suicide ideation (BSS, [28])

The BSS is a 21-item self-report scale that assesses the intensity of current suicidal ideation, with items rated on intensity (range = 0–2). The first 19 items assess suicidal ideation, while the last two items assess past attempts. One item was removed due to item-content overlap with the functions of suicide items described below, and only the items assessing suicidal ideation were used for this study (total of 18 items used). Internal consistency of the 18-item scale was high, with a Cronbach’s α of 0.92 for baseline scores and α = 0.95 for follow-up scores.

#### The suicidal behaviour questionnaire (SBQ-14, [29])

The SBQ-14 is a 34-item self-report scale that assesses suicidal ideation, suicide threats, and likelihood of future suicide attempts. Only two items from the SBQ-14 were used to capture the potential functions of suicidal ideation: (1) *Would any of your problems be solved if you committed suicide?* And (2) *Thinking about the way your life is today, that is, given the good things in your life now and any problems you might be having, IF you knew the QUALITY of your life would never change, that is, it would never get better or worse, do you feel that suicide would be a good way out?* Participants rated their endorsement of these beliefs using a five-point Likert scale, ranging from *no, definitely not* to *yes, definitely*. Intraclass correlation coefficients for the escape item (ICC = .642) and the problem solving item (ICC = .645) between time 1 and time 2 were moderate in size [30].

### Procedures

Participants were invited to take part in a longitudinal study with three assessments, each 6-months apart. For each assessment, participants completed the same series of questionnaires and interviews for 1.5–2 h. Participants were compensated $50 for the first two assessments and $60 for the final assessment. Only data from the first and second assessments were used for this study. Ninety-one percent of participants completed the second assessment on average 7.94 months (*SD* = 2.64 months) following their first assessment. There were no significant differences between participants who dropped out/withdrew and those who completed the second assessment on all study variables (all *ps* > .106).

## Results

SPSS version 24 software was used for all analyses [31]. Means, standard deviations, and intercorrelations for all study variables are included in Table 1. Two subscales of the DERS did not significantly relate to suicidal ideation at baseline or follow-up (Engaging in Goal Directed Behaviour and Lack of Emotional Awareness), therefore moderation effects for these scales were not examined. Assumptions of normality for all study variables were met. Based on the correlation between belief in suicide as an escape and belief in suicide as a solution to a problem (*r* = 0.62), we did not combine these items and tested moderating effect in separate models.

**Table 1:** Means, Standard Deviations, and Intercorrelations of Study Variables

Measure	*M* (SD)	1	2	3	4	5	6	7	8	9	10
1. BSS Baseline	7.77 (7.92)	–									
2. DERS	112.16 (22.00)	.32**	–								
3. DERS1	18.83 (6.29)	.28**	.75**	–							
4. DESR2	17.68 (4.59)	.17	.73**	.41**	–						
5. DERS3	18.24 (5.40)	.29**	.84**	.59**	.70**	–					
6. DERS4	16.12 (4.77)	.18	.25*	−.02	−.10	−.08	–				
7. DERS5	26.37 (6.44)	.25**	.85**	.69**	.63**	.70**	−.08	–			
8. DERS6	14.56 (4.03)	.24*	.61**	.23*	.32**	.35**	.46**	.34**	–		
9. Escape	1.54 (1.32)	.55**	.31**	.16	.10	.26*	.22*	.23*	.27*	–	
10. Problem Solve	1.72 (1.43)	.68**	.31**	.33**	.13	.27**	.11	.20	.26*	.62**	–
11. BSS Follow-up	6.12 (8.32)	.70**	.28*	.27*	.20	.24*	.01	.24*	.21*	.46**	.69**

*Note.* *p < .05, **p < .01. Numbered measures indicate subscales. *BSS* Beck Scale for Suicide Ideation, *DERS* Difficulties in Emotion Regulation, *DERS1* Nonacceptance of Emotional Responses, *DERS2* Engaging in Goal-Directed Behaviour, *DERS3* Impulse Control Difficulties, *DERS4* Lack of Emotional Awareness, *DERS5* Limited Access to Emotion Regulation Strategies, *DERS6* Lack of Emotional Clarity, Escape = Suicidal Behaviour Questionnaire, “Way out” Item. Problem Solve = Suicidal Behaviour Questionnaire, “Problem Solve” Item

### Main analyses

#### Cross-sectional findings

**Fig. 1:**
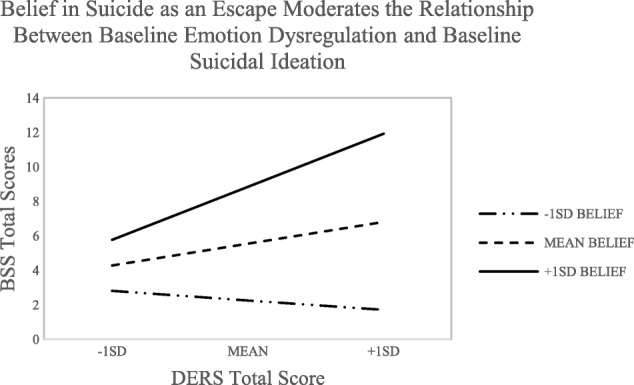
Moderation Effect for Difficulties in Emotion Regulation Scale Total Score*.* BSS = Beck Scale for Suicidal Ideation. DERS = Difficulties in Emotion Regulation Scale. SD = Standard Deviation. Belief scores were obtained from item 29 on the Suicidal Behaviour Questionnaire

Baseline age was significantly related to both emotion dysregulation (*r* = −.36, *p* < .01) and baseline suicidal ideation scores (*r* = −.28, *p* < .01) and gender was significantly related to emotion dysregulation (*r* = .39, *p* < .01), as such both age and gender were included as covariates in all analyses. We used *PROCESS* [32], an add-on macro in SPSS, and 5000 bootstrapped resamples to examine the moderation effect. A hierarchical linear regression was conducted that included emotion dysregulation scores and the function of suicide items as predictors (in two separate models), and suicidal ideation as the outcome variable. To correct for multiple comparisons, all effects were considered significant at the *p* < .01 level. For our initial model, we examined if the total emotion dysregulation score and the function of suicide as an escape predicted suicidal ideation severity. These predictors accounted for a significant amount of the variance in suicidal ideation scores (*R*^*2*^ = 0.40, *F* (5, 71) = 9.51, *p* < .000). To test the moderation effect, we calculated an interaction term between emotion dysregulation and the escape item after centering both variables [33]. The interaction term approached significance (Δ*R*^*2*^ = 0.05, *F* (1, 71) = 6.42, *p* = .013). Johnson-Neyman significance regions were examined and an interaction plot was created (Fig. 1), illustrating that as the belief in suicide as an escape increased, so did the positive relationship between emotion dysregulation and suicidal ideation. When belief in suicide as an escape was low, the relationship between emotion dysregulation and suicidal ideation was non-significant (*ps* > .05).

We examined the predictive power of the total emotion dysregulation score and belief in suicide as a solution to a problem in predicting suicidal ideation severity. Emotion dysregulation and belief in suicide as a solution to a problem predicted a significant amount of variance in suicidal ideation severity (*R*^*2*^ = 0.50, *F* (5, 73) = 14.80, *p* < .000), however our moderator variable did not significantly add to the model (Δ*R*^*2*^ = 0.01, *F* (1, 73) = 0.76, *b* = 0.07, *SE* = 0.08, *t* = 0.87, *p* = .385, 95% CI [− 0.090, 0.231]).

#### Longitudinal findings

Baseline age, gender, and suicidal ideation were included as covariates. Hierarchical linear regression models were conducted that included emotion dysregulation scores and the function of suicide items as predictors (in two separate models), and suicidal ideation at follow-up as the outcome variable. Baseline emotion dysregulation and belief in suicide as an escape predicted follow-up suicidal ideation (*R*^*2*^ = 0.59, *F* (6, 62) = 14.95, *p* < .000), although this belief did not moderate the relationship between baseline emotion dysregulation and longitudinal suicidal ideation (Δ*R*^*2*^ = 0.03, *F* (1, 62) = 4.29, *p* = .043; see Table 2).

**Table 2:** Moderation Effects for the Belief in Suicide as an Escape for Baseline and 6-Month Suicidal Idea*tion*

Moderation effect	*b*	*SE*	*t*	*p*	95% CI
Cross-Sectional Findings					
DERS x Escape	0.26	0.10	2.54	0.013	0.056, 0.466
DERS1 x Escape	0.28	0.10	2.71	0.008	0.073, 0.477
DERS3 x Escape	0.20	0.09	2.22	0.029	0.021, 0.379
DERS5 x Escape	0.21	0.10	1.99	0.050	0.000, 0.410
DERS6 x Escape	0.15	0.10	1.53	0.131	−0.045, 0.342
Longitudinal Findings					
DERS x Escape	0.18	0.09	2.07	0.043	0.006, 0.346
DERS1 x Escape	0.19	0.10	1.09	0.280	−0.090, 0.307
DERS3 x Escape	0.05	0.08	0.59	0.557	−0.113, 0.209
DERS5 x Escape	0.11	0.10	1.09	0.279	−0.087, 0.297
DERS6 x Escape	0.22	0.09	2.56	0.013	0.049, 0.396

*Note.* Numbered measures indicate subscales. *DERS* Difficulties in Emotion Regulation Scale, *DERS1* Nonacceptance of Emotional Responses, *DERS3* Impulse Control Difficulties, *DERS5* Limited Access to Emotion Regulation Strategies, *DERS6* Lack of Emotional Clarity. Escape = Suicidal Behaviour Questionnaire, “Way out” Item. All longitudinal analyses included baseline suicidal ideation as a covariate

We then investigated if baseline belief that suicide is a solution to a problem moderated the relationship between baseline emotion dysregulation and suicidal ideation at follow-up. While the overall model was significant (*R*^*2*^ = 0.65, *F* (6, 63) = 19.70, *p* < .000), this belief did not moderate the emotion dysregulation-suicidal ideation longitudinal relationship (Δ*R*^*2*^ = 0.02, *F* (1, 63) = 3.71, *b* = 0.13, *SE* = 0.07, *t* = 1.93, *p* = .059, 95% CI [− 0.005, 0.026]).

#### Exploratory analyses

**Fig. 2:**
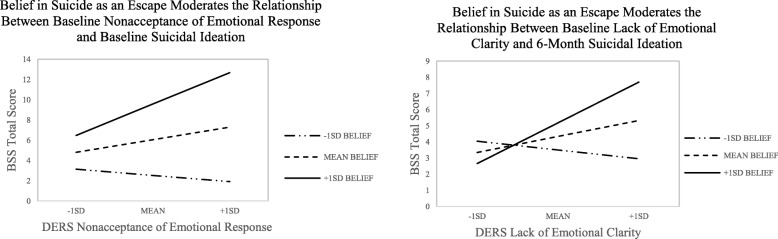
Moderation Effects for Difficulties in Emotion Regulation Subscale Scores*.* BSS = Beck Scale for Suicidal Ideation. DERS = Difficulties in Emotion Regulation Scale. SD = Standard Deviation. Belief scores were obtained from item 29 on the Suicidal Behaviour Questionnaire

We additionally explored the role of these beliefs in moderating the relationships between subscales of emotion dysregulation and suicidal ideation cross-sectionally and longitudinally. We included the same covariates as above for these analyses and considered findings significant at the *p* < .01 level. For our cross-sectional findings, belief in suicide as an escape significantly moderated the relationship between Nonacceptance of Emotional Responses subscale (Δ*R*^*2*^ = 0.06, *F* (1, 78) = 7.33, *p* = .008) and baseline suicidal ideation, while it did not moderate any other relationships (*p* > .029). Johnson-Neyman significance regions were examined for these subscales and interaction plots were created (Fig. 2), illustrating the same pattern that was observed for the total emotion dysregulation scale (see also Table 2). Belief in suicide as a solution to a problem did not moderate any of the relationships between emotion dysregulation subscales and suicidal ideation cross-sectionally (all *ps* > .210).

We then investigated if belief in suicide as an escape moderated the relationship baseline emotion dysregulation subscales and follow-up suicidal ideation. For these models, we included baseline suicidal ideation as a covariate. A trend was found for belief in suicide as an escape moderating the relationship between Lack of Emotional Clarity and suicidal ideation (Δ*R*^*2*^ = 0.04, *F*(1, 73) = 6.56, *p* = .013; see Table 2 and Fig. 2). Because of previous controversy surrounding the inclusion of a covariate that is correlated with the independent variable in a model [34, 35], we also examined the relationship between Lack of Emotional Clarity and suicidal ideation ~ 6-months *without* the inclusion of baseline suicidal thoughts, finding that the model was significant and had more predictive power (Δ*R*^*2*^ = 0.10, *F* (1, 75) = 11.00, *b* = 0.33, *SE* = 0.10, *t* = 3.32, *p* = .001, 95% CI [0.133, 0.534]). Belief in suicide as a solution to a problem did not moderate the relationship between any emotion dysregulation subscales and suicidal ideation at follow-up (all *ps* > .021).

## Discussion

In this study, we explored if beliefs in the functions of suicide moderated the relationship between emotion dysregulation and suicidal ideation in a community sample. Two beliefs were examined: (1) suicide as an escape from emotional pain and (2) suicide as a solution to one’s problem. We also examined this moderation cross-sectionally and longitudinally. For our cross-sectional findings, we found a trend towards belief in suicide as an escape moderating the relationship between global emotion dysregulation and severity of suicidal ideation, while this was not true for belief in suicide as a solution to a problem. For our longitudinal findings, we did not find that either belief moderated the relationship between global emotion dysregulation and longitudinal suicidal ideation.

We also explored how these two beliefs moderated the relationship between facets of emotion dysregulation and suicidal ideation both cross-sectionally and longitudinally. While belief in suicide as a solution to a problem did not moderate any of these relationships, we found that belief in suicide as an escape moderated the relationship between Nonacceptance of Emotional Responses and cross-sectional suicidal ideation and between Lack of Emotional Clarity and longitudinal suicidal ideation (a relationship that approached significance when baseline suicidal ideation was included as a covariate and a relationship that was significant when baseline suicidal ideation was not adjusted for). Our findings suggest that belief in suicide as an escape or a solution to a problem have distinct mechanisms in how they relate to suicidal ideation. This is exemplified by the fact that suicide as a solution to a problem was significantly related to severity of suicidal ideation both cross-sectionally and longitudinally, even though it did not moderate the relationship between emotion dysregulation and suicidal ideation like belief in suicide as an escape did.

### Moderation effects

Recent investigations have found a relationship between general experiential avoidance and suicidal ideation [12, 13], yet no studies have examined how holding the specific belief that suicide is an escape from emotional pain might moderate the relationship between a known risk factor for suicidal ideation (i.e., emotion dysregulation) and the severity of suicidal ideation. While we found that holding the belief that suicide is an escape moderated the relationship between emotion dysregulation and suicidal ideation in the expected direction, what is more interesting is how the emotion dysregulation-suicidal ideation relationship was altered for individuals who did not highly endorse this belief. For these participants, high emotion dysregulation was not related to higher levels of suicidal ideation. This illustrates the importance of addressing underlying beliefs about the function of suicide in treatment in conjunction with addressing emotion dysregulation, and especially takes into consideration the importance of experiential avoidance in maintaining suicidal ideation. While interventions might focus more exclusively on providing emotion regulation strategies to reduce the intensity and frequency of emotion dysregulation, it could prove beneficial to also use cognitive restructuring to address beliefs related to experiential avoidance and nonacceptance of emotional distress. For instance, a client who believes “I can’t handle this” or “it is too much, I need to get out” might benefit from cognitive tools that illustrate previous incidents of being able to manage distress effectively without escape. This client might also benefit from mindfulness strategies that include sitting with distress and tolerating and accepting emotions without following through with urges for avoidance/escape. In this manner, when the client faces moments of emotion dysregulation in the future, they might be less likely to consider suicide as a way to escape momentary distress. Rather, such clients might more easily recall moments of being able to cope with distress (i.e., cognitive restructuring) and might use newly acquired mindfulness skills to sit with distress without avoidance or escape.

We did not find that belief in suicide as an escape moderated the relationship between baseline global emotion dysregulation and suicidal ideation at follow-up. While this may seem to suggest that the moderating effect of this belief is only relevant cross-sectionally, it is also possible that our analyses lacked sufficient power to detect an effect. When not taking multiple comparisons into account, belief in suicide as an escape was found to significantly moderate the relationship between baseline emotion dysregulation and follow-up suicidal ideation. Further, belief in suicide as an escape moderated the relationship between a facet of emotion dysregulation, Lack of Emotional Clarity, and ~ 6-month suicidal ideation. This suggests that this belief might be rigid and long-standing, as it may continue to play an important role in the strength of the relationship between emotion dysregulation and suicidal ideation longitudinally. If true, then belief in suicide as an escape might be a maintaining factor for considering suicide in instances of emotional pain. We recommend that future research explore the moderating role of this belief in the relationship between emotion-dysregulation and follow-up suicidal ideation further and examine if these findings can be replicated.

We also found, in exploratory analyses, that belief in suicide as an escape moderated the relationship between facets of emotion dysregulation and suicidal ideation both cross-sectionally and longitudinally, while belief in suicide as a problem-solving strategy did not. More specifically, belief in suicide as an escape moderated the relationship between Nonacceptance of Emotional Responses and baseline suicidal ideation and Lack of Emotional Clarity and follow-up suicidal ideation. Differences found between moderation effects for cross-sectional and longitudinal associations might be due to limited power in our longitudinal associations. It could also be that current nonacceptance of emotional pain results in an immediate spike in emotional suffering, which leads individuals who believe suicide is a way out to experience greater current severity of suicidal ideation. On the other hand, a general lack of clarity in emotional responses might lead to greater emotional suffering over time and therefore predict suicidal ideation severity ~ 6 months later for individuals who strongly endorse the belief that suicide is an escape at baseline. It is interesting to note that the effect sizes based on standardized coefficients for facets of emotion dysregulation were generally higher than those for total emotion dysregulation scores, although it is unclear if this represents a statistically significant difference. This might point to the importance of exploring the influence of beliefs on emotion dysregulation primarily at the facet level. Future research should endeavor to examine these beliefs and their impact on facets of emotion dysregulation while also considering differing time-scales, including longitudinal assessment and day-to-day examination of these constructs using experience sampling approaches.

While we did not find that the belief that suicide is a solution to a problem moderates the relationship between emotion dysregulation (both global scores and facets of emotion dysregulation) and suicidal ideation severity, this belief was associated with the severity of suicidal ideation both at baseline and at follow-up. This would suggest that each belief might be relevant in distinct contexts. The belief that suicide is a solution to one’s problem might be related to long-term risk factors instead of an inability to regulate emotional pain, such as chronic life stress and chronic pain. This would suggest that intervention strategies for addressing the belief that suicide as a solution to a problem might focus less on emotion regulation skills and more on behavioural principles of problem-solving (e.g., brainstorming, pros/cons), in conjunction with cognitively restructuring beliefs surrounding one’s own problem-solving abilities (e.g., “I am a terrible problem-solver”). It should be noted that the belief that suicide is a solution to a problem approached significance for the Lack of Emotional Clarity facet of emotion dysregulation in our longitudinal analyses. It is recommended that future research continue to examine the potential impact of holding the belief that suicide is a solution to a problem on the severity of suicidal ideation and its relationship with facets of emotion dysregulation.

#### Limitations and future directions

There are some important limitations to consider. Our assessment of the beliefs about suicide were examined using single-item responses instead of full scales. The use of single-item measures is considered problematic by some researchers (e.g., [36]). It should be noted, however, that previous investigations have illustrated that single-item measures are adequate for simple and homogenous constructs, while they are problematic for more complex and heterogeneous ones [37]. Others have found no significant differences in validity or reliability between single and multiple-item measures examining similar phenomena [38, 39]. Further, our intraclass correlation values fall in the moderate range for items that have been recorded at minimum 6-months apart, suggesting that the items are reliable. We were unable to use multiple-item measures because such measures do not yet exist for these constructs. We recommend that future research use multiple-item measures.

The sample in this study was recruited from the community instead of using a clinical population. Although some might consider this a limitation, it is clear that our sample exhibited scores similar to clinical samples for our emotion dysregulation and suicidal ideation scales. The mean emotion dysregulation score in this sample was 112.16 (*SD* = 22.00) which is comparable to a recent study showing a mean score of 109.73 (*SD* = 24.95) in an outpatient sample seeking dialectical behaviour therapy [40]. The mean baseline suicidal ideation score in this sample was 7.77 (*SD* = 7.92) which is also comparable to a mean of 8.42 (*SD* = 10.26) in a mixed inpatient/outpatient sample [28], although our mean follow-up suicidal ideation score was lower (*M =* 6.12, *SD* = 8.32). Based on our mean sample scores, it is clear that our participants exhibited similar levels of psychopathology as seen by their clinical counterparts.

When considering multiple comparisons, only some of our effects remained significant including the cross-sectional moderation for the relationship between Nonacceptance of Emotional Response and suicidal ideation and the longitudinal relationship between baseline Lack of Emotional Clarity and follow-up suicidal ideation (only if baseline suicidal ideation was not included as a covariate in the model). We therefore suggest that findings pertaining to total emotion dysregulation scores be seen as tentative and in need of future verification.

## Conclusions

In this study, we examined two beliefs about the function of suicidal ideation that are related and yet were found to have distinct effects on the relationship between emotion dysregulation and suicidal ideation. More specifically, believing that suicide is an escape from pain altered the strength of the emotion dysregulation-suicidal ideation relationship while believing suicide is a solution to a problem did not. It is important to note that this does not suggest that only some cognitive factors are important in predicting suicidal ideation, yet it illustrates the complexity of each belief and its differing impact on vulnerability factors found to predict suicidal ideation. Findings from this study also illustrate the importance of not only addressing suicidal ideation vulnerability factors, such as emotion dysregulation, but also examining and changing underlying beliefs about the function of suicidal ideation, especially in considering their impact on longitudinal suicidal thoughts. After an assessment of underlying beliefs about suicide, clinicians can choose alternative strategies to address these beliefs more readily. This study is the first to explore the moderating role of beliefs about suicide in the relationship between emotion dysregulation and suicidal ideation severity, illustrating the importance of considering cognitive factors when assessing suicidal ideation. Replication is important to further substantiate these findings.

## Data Availability

The dataset used and/or analyzed during the current study are available from the corresponding author on reasonable request.
